# The P-Loop Domain of Yeast Clp1 Mediates Interactions Between CF IA and CPF Factors in Pre-mRNA 3′ End Formation

**DOI:** 10.1371/journal.pone.0029139

**Published:** 2011-12-22

**Authors:** Sandra Holbein, Simonetta Scola, Bernhard Loll, Beatriz Solange Dichtl, Wolfgang Hübner, Anton Meinhart, Bernhard Dichtl

**Affiliations:** 1 Centre for Cellular and Molecular Biology, School of Life and Environmental Sciences, Deakin University, Burwood, Australia; 2 Institute of Molecular Life Sciences, University of Zürich, Zürich, Switzerland; 3 Fachbereich Biologie, Chemie, Pharmazie, Institut für Chemie und Biochemie, AG Strukturbiochemie, Freie Universität Berlin, Berlin, Germany; 4 Structural and Computational Biology, EMBL Heidelberg, Heidelberg, Germany; 5 Department of Biomolecular Mechanisms, Max Planck Institute for Medical Research, Heidelberg, Germany; The John Curtin School of Medical Research, Australia

## Abstract

Cleavage factor IA (CF IA), cleavage and polyadenylation factor (CPF), constitute major protein complexes required for pre-mRNA 3′ end formation in yeast. The Clp1 protein associates with Pcf11, Rna15 and Rna14 in CF IA but its functional role remained unclear. Clp1 carries an evolutionarily conserved P-loop motif that was previously shown to bind ATP. Interestingly, human and archaean Clp1 homologues, but not the yeast protein, carry 5′ RNA kinase activity. We show that depletion of Clp1 in yeast promoted defective 3′ end formation and RNA polymerase II termination; however, cells expressing Clp1 with mutant P-loops displayed only minor defects in gene expression. Similarly, purified and reconstituted mutant CF IA factors that interfered with ATP binding complemented CF IA depleted extracts in coupled *in vitro* transcription/3′ end processing reactions. We found that Clp1 was required to assemble recombinant CF IA and that certain P-loop mutants failed to interact with the CF IA subunit Pcf11. In contrast, mutations in Clp1 enhanced binding to the 3′ endonuclease Ysh1 that is a component of CPF. Our results support a structural role for the Clp1 P-loop motif. ATP binding by Clp1 likely contributes to CF IA formation and cross-factor interactions during the dynamic process of 3′ end formation.

## Introduction

In eukaryotes, processing of primary transcripts is essential for RNAs to acquire biological function. Maturation of mRNAs includes formation a 7-methyl-guanosine cap modification at the 5′ end and the addition of a poly(A) tail to the 3′ end. Maturation at the 3′ end affects the release of the RNA from the site of transcription, the efficiency of translation and the susceptibility to nucleolytic degradation [1]. The process of 3′ end formation is functionally linked to transcription by RNA polymerase II and involves dynamic interactions of processing factors with the CTD domain of the polymerase [2].

Pre-mRNA 3′ end formation is initiated by endonucleolytic cleavage at the poly (A) site, which is tightly coupled to polyadenylation of the upstream cleavage product [1], [3]. In yeast, activities associated with cleavage factor IA (CF IA), cleavage factor IB (CFI B), and cleavage and polyadenylation factor (CPF) factors are sufficient to reconstitute both steps of the reaction *in vitro* [1], [3]. The protein complement of this complex machinery has been well characterized: CFI B consist of a single subunit Hrp1 [4], [5] and CF IA is comprised of the four subunits Rna14, Rna15, Pcf11 and Clp1 [6], [7], [8]. CPF consists of some fifteen subunits [9] and several sub-complexes of this factor have been characterized: Cleavage factor II (CF II) [10], which consists of Yhh1/Cft1, Pta1, Ydh1/Cft2, and the 3′ endonuclease Ysh1/Brr5 [11], [12]; polyadenylation factor I (PF I), which contains all CF II subunits, the poly(A) polymerase Pap1, Pfs1, Pfs2, Fip1 and Yth1 [13]; and the APT sub-complex in which Pti1, Ref2, Syc1, Swd2, Ssu72, Glc7 associate with PF I via the Pta1 subunit [14].

The yeast Clp1 protein (which will be referred to solely as ‘Clp1’ for the remainder of this manuscript, while homologous proteins from other organisms will carry a prefix), a subunit of CF IA, is encoded by an essential gene that has evaded detailed study to date. Structural analysis of Clp1 in association with a fragment of its CF IA interaction partner Pcf11 revealed a WalkerA or P-loop domain followed by switch I and switch II domains in the central part of the protein [15]. Clp1 was found to be associated with ATP but attempts failed to demonstrated ATP hydrolysis by the protein [15].

Several homologues of yeast Clp1 have been previously characterized including human [16], plant [17] and archaeal proteins [18]. In human cells, hClp1 is part of the mammalian cleavage factor II (CF II_m_) complex along with hPcf11 [16]. Immunodepletion of hClp1 in HeLa cells eliminated cleavage activity but had no effect on polyadenlyation [16]. Through assays of RNA kinase activity hClp1 has been shown to have 5′ OH polynucleotide kinase activity with a preferences for RNA compared to DNA [19], [20]. hClp1 was able to complement mutations in the RNA kinase module of tRNA ligases from yeast and plant that act during tRNA splicing but it remains unclear whether the protein plays a similar role in human cells [20]. In archaea characterisation of *Pyrococcus horikosii* Clp1 showed that it too is a 5′OH polynucleotide kinase that can perform the kinase step during yeast tRNA splicing [18]. Despite their structural and sequence similarities hClp1 can not rescue lethality associated with a deletion of the *CLP1* locus in yeast [20]. This evidence taken along with the fact that Clp1 has no detectable RNA kinase activiy *in vitro* [20] and that mutations in ATP-binding site of Clp1 do not affect yeast viability, it would seem that hClp1 and Clp1 are not functional orthologs [20].

Here, we analyzed the role of Clp1 during the process of pre-mRNA 3′ end formation. Consistent with the notion that the protein is an essential component of the CF IA factor, depletion of Clp1 in cells caused defective 3′ end formation and transcriptional read-through. However, the P-loop motif and ATP binding appear to play only a minor role for these functions of Clp1. Our results support a structural role for ATP binding to Clp1, which promotes protein interactions required for assembly of the CF IA complex and which contributes to cross-factor interactions during pre-mRNA processing.

## Results and Discussion

### Cellular depletion of Clp1 interferes with 3′ end formation and RNAP II transcription termination

**Figure 1:**
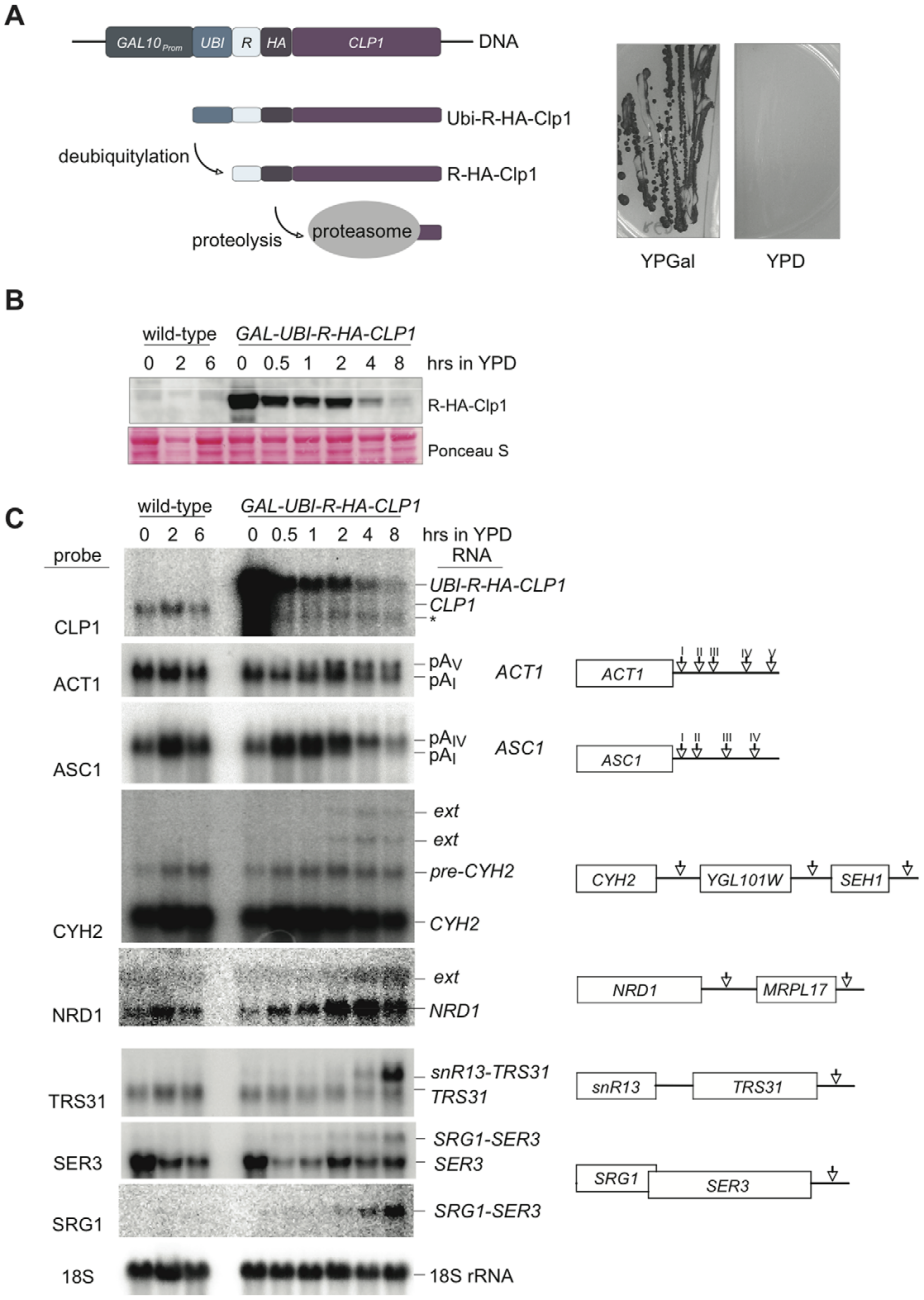
Effects of Clp1 depletion on gene expression. (A) Schematic presentation of the Clp1 fusion gene, which is expressed under the control of *GAL10* promoter. Encoded are an amino-terminal ubiquitin moiety fused in frame to Clp1 together with an HA-tag. Removal of the ubiquitin moiety by cellular deubiquitylases results in a protein with an arginine (R) at the amino-terminus creating an unstable protein that is targeted for degradation by the proteasome. A strain carrying this fusion gene is viable on YPGal medium but not on YPD, which contains repressive glucose as sole carbon source. (B) Western Blot of total extracts obtained from wild-type and *GAL-UBI-R-HA-CLP1* expressing strains following growth in YPD for the indicated times. Western blots were decorated with antibody against the HA-tag. Ponceau S staining served as control for equal loading. (C) Northern analysis of total RNAs obtained from wild-type and *GAL-UBI-R-HA-CLP1* expressing strains following growth in YPD for the indicated times. RNAs were detected with random prime labeled probes directed against the open reading frames as indicated on the left of each panel. The asterisk in the first panel marks a likely degradation product of the *UBI-R-HA-CLP1* mRNA. In *CYH2* and *NRD1* panels ‘ext’ denotes 3′ extended transcripts. 18S rRNA was detected with an end-labeled oligonucleotide and was used as control for equal loading. Schematically depicted on the right are features of analyzed and neighbouring genes with arrows indicating sites of 3′ end cleavage and polyadenylation.

Yeast Clp1 is known to be involved in 3′end processing but its precise role remained unclear [7], [21]. We sought to deplete the protein from cells and to determine the resulting effects on gene expression. Since Clp1 is essential for vegetative growth we chose to construct a strain in which the protein could be conditionally depleted. Figure 1A outlines a plasmid-borne construct, which was expressed in haploid strains lacking the chromosomal copy of the *CLP1* gene. In the presence of galactose an in-frame fusion of ubiquitin, the HA-epitope tag and Clp1 was expressed (Ubi-R-HA-Clp1) [22], [23]. Cleavage of the ubiquitin moiety through cellular deubiquitylating enzyme generated protein that carried an arginine at its amino-terminus and thus, according to the N-end rule, rendered the protein unstable [22]. Strains expressing *GAL-UBI-R-HA-CLP1* grew without apparent growth defect on galactose medium indicating that the Ubi-R-HA-Clp1 fusion protein was functional (Fig. 1A). In contrast, no growth of the strain was observed in the presence of glucose (YPD), indicating that the protein was efficiently depleted. This was verified by Western blotting using antibodies directed against the HA-tag. Figure 1B shows a strong decrease in Ubi-R-HA-Clp1 protein following growth in YPD for four hours and almost complete depletion after eight hours.

To determine the effects of Clp1 depletion on gene expression, different classes of RNA Pol II transcripts were analyzed by Northern blot (Fig. 1C). We initially probed for expression of the *UBI-R-HA-CLP1* transcript that was over-expressed in galactose (0 hrs in YPD) and strongly reduced after shift to YPD (4 and 8 hrs). In the wild-type expression of the shorter *CLP1* mRNA was detected, which was unaltered in the presence of galactose. The *ACT1* gene carries a cluster of alternative poly (A) sites in its 3′UTR [24]. In wild-type strains the most proximal and stronger pA site is used predominantly but mutations within 3′ end processing factors [25] or the chemical inhibitor cordycepin [26] promote usage of the distal pA-sites. Similarly, Clp1 depletion correlated with an accumulation of longer transcripts that resulted from the use of distal pA-sites. The intron-containing *ASC1* gene also contains alternative poly(A) sites [26]. While *ASC1* mRNA levels were strongly reduced after eight hours YPD, preferred usage of more distal poly(A) sites was apparent at earlier time points. The intron containing *CYH2* gene does not carry closely spaced alternative poly(A) sites, but *CYH2* mRNAs are strongly extended in the presence of the 3′ end formation inhibitor cordycepin [26]. Similarly, we observed extended *CYH2* RNAs following Clp1 depletion. Pre-mRNA splicing of *CYH2* transcripts is slow allowing for ample accumulation of the unspliced precursor [27]. Stable levels of pre-*CYH2* following Clp1 depletion suggested that the protein was not required for pre-mRNA splicing. Finally, we looked at RNAP II transcripts that are expressed in a Nrd1-dependent pathway [28]. Nrd1p controls its own expression through regulated premature termination [29]. As Clp1 becomes depleted an increase in *NRD1* transcripts was observed, suggesting that Nrd1 auto-regulation was defective. Furthermore, we observed the accumulation of extended *NRD1* transcripts, which likely corresponded to *NRD1-MRPL17* read-through transcripts [28], [30]. Transcriptional read-through was also detected at the snR13 locus resulting in strong accumulation of extended snR13-TRS31 RNAs after eight hours in YPD. Finally, we observed read-through at the terminator for the *SRG1* cryptic unstable transcript (CUT) resulting in the accumulation of bicistronic *SRG1-SER3* RNAs. Taken together, our results show that depletion of Clp1 resulted in defective expression of all analyzed RNAP II transcription units. Both poly(A)-dependent and poly(A)-independent 3′ end formation pathways were affected resulting in deficient transcriptional termination. Interestingly, both of these pathways also require Pcf11, whereas this remains unclear for the CF IA components Rna14 and Rna15 [31], [32]; our observations point towards a possible requirement for a functional Pcf11-Clp1 dimer at polyA dependent and independent terminators.

### Clp1 mediates protein interactions with CF IA and CPF

**Figure 2:**
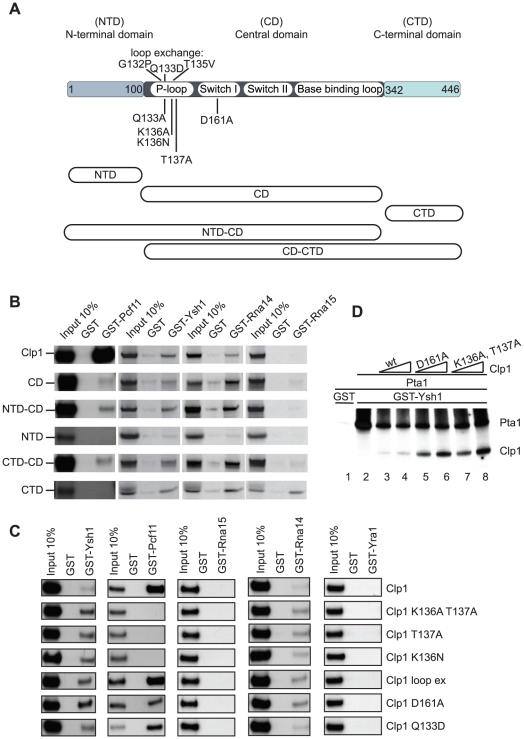
Clp1 mediates interactions between CF IA and CPF factors. (A) Schematic presentation of the yeast Clp1 domain structure. Indicated are N-terminal domain (NTD), central domain (CD) and the C-terminal domain (CTD), as well as the locations of mutations analyzed in this study. The lower part of the panel shows Clp1 fragments, which were analyzed in binding studies. (B) GST pull-down experiments with GST, GST-Pcf11, GST-Ysh1, GST-Rna14 and GST-Rna15 and *in vitro* translated and [^35^S]-methionine-labeled proteins as indicated on the left. Input shows 10% of total radioactive material included in the binding reactions. (C) GST pull-down experiments using the indicated GST-fusion proteins and *in vitro* translated and [^35^S]-methionine-labeled full length wild-type and mutant Clp1 proteins as indicated on the right. (D) GST pull-down experiment with GST-Ysh1 and *in vitro* translated and [^35^S]-methionine-labeled proteins. All binding reactions contained labeled Pta1 as well as increasing concentrations of wild-type and mutant Clp1 proteins as indicated. On separate gels (not shown) it was ensured that equivalent amounts of radioactive protein was included in reactions where binding of wild-type Clp1 protein was compared to D161A and K136A T137A mutants.

Previous work revealed that Clp1 binds to the Pcf11 subunit of CF IA and the Ysh1 subunit of CPF [21], [25]. To further understand the role of Clp1 in 3′end formation we wished to determine the domains that were mediating interactions. For this purpose we dissected the role of three Clp1 domains (Fig. 2A): a N-terminal domain (NTD; amino-acids 1 to 100), a central domain (CD; amino-acids 101 to 341) and a C-terminal domain (CTD; amino-acids 342 to 446). The central domain contains the P-loop motif, the switch I and switch II motif as well as the base binding loop, which are involved in nucleotide binding [15]. Pull-down experiments were performed with GST-fusion proteins and *in vitro* translated, ^35^S-labelled full-length Clp1 or fragments encompassing CD, NTD-CD, CTD-CD, NTD, or CTD domains (Fig. 2B). We found that GST-Pcf11 bound strongly to full-length Clp1 and weakly to isolated Clp1 domains. The latter weak interactions were observed with fragments containing the CD but not with NTD and CTD peptides. These results suggested that efficient binding to Pcf11 required full-length Clp1 and that the CD was involved in the interaction between these two CF IA subunits. In contrast, GST-Ysh1 and GST-Rna14 bound to all CD-containing domains of Clp1 and the CTD but not to the NTD. Thus, the interactions between Ysh1, Rna14 and Clp1 involve both the CD and the CTD. With GST-Rna15 significant pull-down was only observed for the CTD fragment. Since no binding was detected with full-length the relevance of this *in vitro* interaction remains unclear. Taken together, these results revealed that the Clp1-CD is involved in mediating interactions with subunits of both CF IA and CPF.

Since the P-loop motif of Clp1 is contained within the CD domain we wished to determine whether an intact P-loop was required for binding to Pcf11 and Ysh1. For this purpose GST pull-down experiments were performed with wild-type and mutant Clp1 proteins (Fig. 2C). Specifically, we tested single mutations within the P-loop motif (Q133D, K136N, T137A) and the switch I region (D161A), the double mutation K136A T137A and the triple mutation loop exchange (G132P Q133D T135V); the latter mutations reconstitute amino acids of the hClp1 P-loop motif within the yeast protein. We found that binding to GST-Pcf11 was completely lost with K136A or T137A single mutations and K136A T137A double mutations whereas binding efficiency was compromised with the switch I mutation D161A. In contrast, binding was efficient with Q133D and loop exchange mutants.

Interestingly, GST-Ysh1 interacted with all tested Clp1 proteins (Fig. 2C). However, in multiple experiments binding was consistently weaker with wild-type Clp1 compared to any of the tested Clp1 mutants. This effect was enhanced when binding reactions also contained the CPF subunit Pta1, which directly binds to GST-Ysh1 (Fig. 2D, lane 1). Under these conditions the weaker binding of wild-type Clp1 (lanes 3 and 4) appeared aggravated compared to D161A (lanes 5 and 6) and K136A T137A Clp1 variants (lanes 7 and 8). Together these data suggest that mutations within the Clp1 P-loop caused structural changes that enhanced binding to the 3′ endonuclease Ysh1.

Next, we extended these interaction studies to the remaining CF IA subunits Rna14 and Rna15 (Fig. 2C). While no interactions could be detected between Clp1 proteins and GST-Rna15, binding prevailed with GST-Rna14. Notably, interaction signals in the latter experiments were relatively weak and we found, similarly to the experiments with GST-Ysh1, that the mutant Clp1 proteins bound slightly better compared to the wild-type. Finally, we tested interactions with the mRNP export factor Yra1, which was recently found to bind to Pcf11 adjacent to the Clp1 interaction site [33]. However, no interactions were detected between Clp1 proteins and GST-Yra1.

Our interaction studies suggested that the central domain of Clp1, which included the P-loop motif, modulated binding to 3′ end factors Pcf11, Ysh1 and Rna14. To test whether ATP binding by the P-loop played a role in these interactions we chose to assemble CF IA entirely from heterologously expressed subunits and to test the nucleotide binding state of Clp1 in the context of the reconstituted factors. In these experiments we first immobilized GST-Pcf11ΔN288 (lacking the amino-terminal domain that mediates binding to the RNAP II CTD) on glutathione sepharose and added His6-Clp1 and preformed His6-Rna14/Rna15 dimers. Reconstitution occurred efficiently when all four CF IA subunits were present [34]. In contrast, we were not successful in isolating CF IA factor when Clp1 protein was omitted suggesting that Clp1 was required for assembly of CF IA with recombinant subunits *in vitro* (data not shown).

Next, we wished to produce recombinant CF IA factors with mutant Clp1 proteins that did not interact with Pcf11 in pull-downs (see Fig. 2C). However, attempts to over-express and purify CF IA carrying Clp1 mutated at single positions K136A, T137A, and D161A or at both K136A and T137A, failed due to low solubility of proteins in *E. coli* (data not shown). We speculate that compromised ATP-binding capacity caused aberrant protein folding and aggregation. In contrast, we obtained CF IA carrying wild-type Clp1, Clp1 Q133D and the loop exchange triple mutant (data not shown). We used the reconstituted factors to determine the presence and identity of bound nucleotides. For this purpose protein samples were precipitated and applied to a ProntoSIL C18 reverse phase column. The column was developed with 50 mM potassium phosphate buffer and nucleotide elution was monitored by measuring absorption at 254 nm (see Materials and Methods). These analyses revealed that 57% of the obtained CF IA containing wild-type Clp1 was ATP bound, 43% was nucleotide free and none was bound to ADP (Table 1). The factor carrying the Q133D mutation distributed in comparably sized nucleotide-free, ATP- and ADP-bound fractions. Interestingly, the loop exchange mutant showed no detectable amounts of ATP, while 47% of the samples were ADP bound and 53% did not contain any nucleotide. It remains unclear why no ATP was associated with CF IA carrying the loop exchange mutant. Since we could not detect ATPase activity associated with this factor (data not shown) we suspect that the introduced mutations changed the nucleotide binding preference from ATP to ADP. Irrespective of the underlying reasons, the loop exchange mutant provided us with a source of CF IA that was devoid of ATP and which could be functionally tested using *in vitro* transcription/processing reactions (see below). The observation that the loop exchange mutant efficiently bound to GST-Pcf11 (see Fig. 2C) and readily assembled into recombinant CF IA factor provided strong evidence that the presence of ATP binding was not essential for the interaction between Clp1 and Pcf11 protein. Likewise, interactions with Ysh1 and Rna14 are not expected to depend on the presence of ATP since interactions occurred with the loop exchange mutant (Fig. 2C). We interpret these data such that ADP, which was the only nucleotide found associated with this mutant, can functionally replace ATP to support interaction with Pcf11, Ysh1 and Rna14. Alternatively, the nucleotide may be entirely dispensable for Clp1 to engage in these interactions.

**Table 1:** ATP binding status of recombinant CF IA factors.

CF IA factor	nucleotide free	ATP	ADP
wild-type	43%	57%	-
Clp1 Q133D	38%	33%	29%
Clp1 G132P Q133D T135V (loop exchange)	53%	-	47%

### Gene expression in *CLP1* ATP-binding mutants

**Figure 3:**
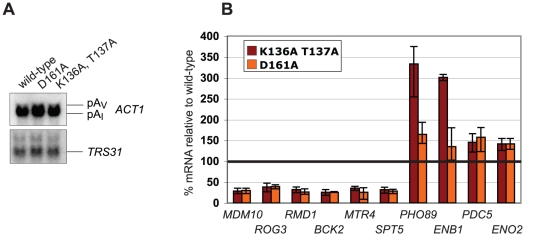
Gene expression in strains expressing Clp1 proteins carrying a mutant P-loop. (A) Northern analysis of total RNAs obtained from wild-type and *CLP1* D161A and *CLP1* K136A T137A mutant strains following growth in YPD. Probes were as described in the legend of figure 1C. (B) qRT-PCR analysis of candidate mRNAs that were identified during gene expression profiling of *CLP1* D161A and *CLP1* K136A T137A mutant strains. Transcript levels are presented relative to wild-type *CLP1*, which was fixed at 100%. Data shown are the mean of three independent biological replicates and error bars indicate standard deviation.

We have shown that Clp1 depletion promoted defects in 3′end formation at different classes of RNAP II transcripts (Fig. 1C). These observations raised the possibility that ATP binding by Clp1 may be required for efficient pre-mRNA processing. To investigate this we initially used Northern blotting to analyze gene expression in haploid yeast strains carrying chromosomal deletions of the *CLP1* gene that were complemented either with a plasmid-borne copy of wild-type *CLP1* or with alleles carrying the D161A single and the K136A T137A double mutations. In contrast to the depletion experiments shown in Figure 1 this approach allowed to specifically address the role of the P-loop in gene expression. Remarkably, no effects could be observed in the expression of *ACT1* and *TRS31* mRNAs (Fig. 3A) suggesting that the Clp1 P-loop motif and ATP binding, respectively, were not essential for poly(A) dependent and poly(A) independent 3′ end formation pathways. We extended these analyses and performed genome-wide expression profiling using microarrays and total RNA obtained from the same strains that were used in Figure 3A, i.e. wild-type, the D161A single and the K136A T137A double mutants. Consistent with the absence of apparent growth defects associated with the mutants [15] (data not shown), these experiments suggested that overall gene expression in the analyzed *CLP1* mutants was comparable to wild-type (S.H. and B.D. unpublished results). We identified less than fifty candidate genes that were changed in their expression at least two-fold in both mutants (S.H. and B.D. unpublished results). No significant gene ontology terms could be associated with these genes indicating that no specific class of genes was dependent on an intact *CLP1* P-loop motif (S.H. and B.D. unpublished results). We performed qRT-PCR on selected candidate mRNAs that were decreased or increased in the mutants, respectively. Figure 3B shows that levels of five tested candidate mRNAs were reduced approximately 2-fold. The levels of two analyzed mRNAs (*PHO89* and *ENB1*) were significantly elevated in the K136A T137A strain but less so in the D161A mutant. Taken together, our experiments revealed that mutations within the *CLP1* P-loop motif that are expected to interfere with ATP binding [20] and which diminish the interaction with Pcf11 (Fig. 2C) resulted in minor changes in the expression of a small number of genes.

### An intact Clp1 P-loop is dispensable for 3′ end formation *in vitro*

**Figure 4:**
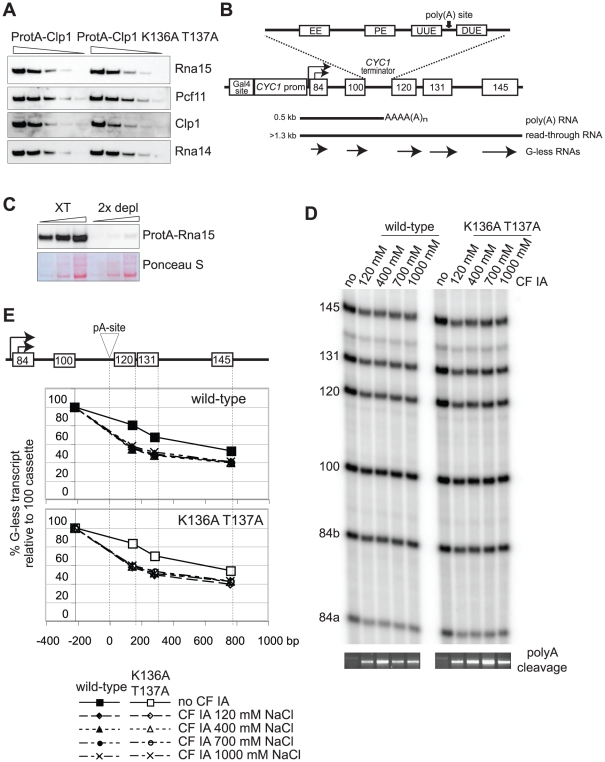
Coupled *in vitro* transcription/3′ end processing. (A) Western blot analysis of CF IA factors partially purified from strains expressing ProtA-Clp1 and ProtA-Clp1 K136A T137A, respectively. Factor purification included a high salt wash (1 M KCl) of bound material to probe stability and integrity of the protein complexes. Decreasing amounts of CF IA associated with ProtA-Clp1 or ProtA-Clp1 K136A T137A were analyzed for the presence of the four CF IA subunits Rna15, Pcf11, Clp1 and Rna14 as indicated on the right. (B) Schematic presentation of the transcription template that was used in transcription/3′ end processing reactions *in vitro*. The construct includes Gal4 binding sites, a *CYC1* promoter and five G-less cassettes; the length of the cassettes in nucleotides is indicated. The *CYC1* terminator has been inserted between the 100 and 120 cassettes; also indicated are specific sequence elements (EE: efficiency element; PE: positioning element; UUE: upstream U-rich element; DUE: downstream U-rich element; and the poly(A) site). *In vitro* transcription produces a 0.5 kb polyadenylated RNA and read-through transcription produces a RNA of more than 1.3 kb length. (C) Western analysis of ProtA-Rna15 expressing whole cell extracts before and after two consecutive rounds of depletion on IgG-agarose. Increasing amounts (1, 2 and 4 µl) of extract (XT) and of depleted extract (2× depl) were resolved by SDS-PAGE and following transfer on PVDF membrane ProtA-Rna15 was detected using an anti-HA-HRP secondary antibody that readily bound to the ProteinA moiety of the fusion protein. Ponceau S staining of a section of the membrane is shown to demonstrate comparable loading between extract and depleted extract. (D) *In vitro* transcription/3′ end processing reactions with CF IA depleted extracts in with or without adding back ProtA-Clp1 and ProtA-Clp1 K136A T137A purified CF IA factors as indicated. On the top of each panel is indicated the salt concentration (mM KCl) applied in the final wash step of the protocol that was applied to purify CF IA factors associated with ProtA-tagged wild-type and mutant Clp1. G-less transcripts were separated on 8.3 M Urea 6% polyacrylamide gels. Migration of the G-less transcripts is indicated on the left. Gels at the bottom of each panel show the analysis of pre-mRNA 3′ end cleavage. Non-radioactive *in vitro* transcription reactions were performed and obtained RNAs were subjected to a ligation-mediated RT-PCR procedure. Formation of PCR product correlates with 3′ end cleavage activity and encompasses the site of poly(A) addition. (E) Quantification of the gels shown in (D). The obtained signals were normalized to uridine-content of the cassettes and presented as percentage of G-less transcription relative to the 100 cassette that is placed immediately upstream of the *CYC1* terminator; the site of poly(A) addition has been fixed at “0” bp. Termination and 3′ end formation is reflected by strength of transcription downstream of the terminator, and is indicated by 120/100, 131/100 and 145/100 signal ratios.

To further test the role of the Clp1 P-loop and ATP binding we used a coupled *in vitro* transcription/processing system based on yeast whole cell extracts [34]. Figure 4B depicts the analyzed transcription unit that carries five G-less cassettes that are increasing in their length in 5′ to 3′ direction of the derived RNA [34]. This construct gives rise to 84a, 84b and 100 nucleotide G-less transcripts that are encoded upstream of the *CYC1* terminator and 120, 131 and 145 nucleotide G-less transcripts that result from transcription downstream of the terminator [34]. Extracts were depleted of endogenous CF IA and defects in transcription termination and in 3′ end formation were rescued by adding back CF IA that was reconstituted from recombinant protein or that was purified from yeast, respectively [34].

Limitations in protein solubility did not allow us to assemble recombinant CF IA with Clp1 protein carrying the K136A T137A mutations (see above). Therefore, we chose to express the protein in yeast fused to a ProteinA-tag and to partially purify the associated CF IA activity. Figure 4A shows that ProteinA-Clp1 K136A T137A co-purified with other CF IA subunits in a stoichiometry that was comparable with wild-type ProteinA-Clp1. Notably, the purification protocol included a final high salt wash step (1 M KCl); thus, a compromised Clp1-Pcf11 interaction as observed with the Clp1 K136A T137A mutant (Fig. 2C), did not significantly disrupt the integrity of CF IA.

Next, we used a coupled *in vitro* transcription/3′ end processing assay [34] to analyze the activity of ProtA-purified CF IA factors carrying wild-type or K136A T137A Clp1 proteins. To resolve potential differences in factor activity resulting from differential stability or purity, respectively, we used increasing amounts of KCl during the final wash steps of affinity purification. A typical experiment is shown in figure 4D and quantification of the results is shown in figure 4E. Depletion of CF IA from extracts resulted in enhanced transcriptional read-through passed the *CYC1* terminator increasing the levels of 120, 131 and 145 G-less cassettes relative to the 100 cassette that is placed just 5′ of the terminator [34]; ‘no CF IA’ in figure 4D and 4E). Add-back of purified factors rescued these phenotypes to the same extend for CF IA containing wild-type and K136A T137A mutant Clp1 (Fig. 4D and 4E). In addition to these transcriptional analyses we also tested 3′ end cleavage during the *in vitro* transcription/processing reactions using a ligation-mediated RT-PCR approach [35]. As expected, the extracts depleted of CF IA had compromised cleavage activity as evidenced by the absence of RT-PCR product (poly(A) cleavage panel in figure 4D). Add-back of wild-type and K136A T137A factors to the reactions rescued this defect showing that 3′ end cleavage occurred efficiently with the mutant factor. To analyze the accuracy of 3′ end cleavage we performed DNA sequencing of ten individual RT-PCR products encompassing the sites of poly(A) addition on transcripts obtained from wild-type and mutant CF IA supplemented reactions. In all cases processing occurred at the major *CYC1* poly(A) site demonstrating that the K136A T137A mutant had no significant impact on poly(A) site recognition and selection (data not shown). We were concerned that the 40 min endpoints of our *in vitro* reactions were not suitable to resolve potential kinetic delays in either transcription termination or 3′ end cleavage that may occur with the mutant factor. To test that we performed time-course experiments analyzing time-points as short as 2.5 min reaction time (data not shown). However, also these conditions did not reveal significant differences in the ability of wild-type and K136A T137 CF IA to rescue defects in transcription termination and 3′ end processing (data not shown).

Our result suggested that a compromised P-loop motif did not interfere with the function of Clp1 in pre-mRNA 3′ end formation and termination *in vitro*. To address more directly the requirement of Clp1 ATP-binding for 3′ end formation we took advantage of the reconstituted CF IA factor that carried the Clp1 loop exchange protein and which we found to carry ADP but not ATP (Table 1). *In vitro* transcription/3′ end processing experiments and 3′ end cleavage analyses were performed with reconstituted CF IA factors as described above for ProtA-purified factors. Complementation of CF IA depleted extracts was found to be equally efficient when recombinant CF IA containing wild-type or loop exchange variants of Clp1 were present (data not shown).

In summary, our *in vitro* analyses suggested that neither an intact Clp1 P-loop, nor ATP binding by CF IA, respectively, were essential for transcription termination and pre-mRNA 3′ end formation *in vitro*. Since mutations interfering with the P-loop structure (K136A T137A and D161A) had only marginal effects on gene expression *in vivo* we consider it unlikely that ATP hydrolysis represents an essential functional role for Clp1 in its association with CF IA. Our protein-interaction studies revealed, however, that the P-loop mutations disrupted the interaction between Clp1 and Pcf11 (Fig. 2C). Since we were not able to assign defects in pre-mRNA processing or transcription termination to the K136A T137A containing CF IA factor we conclude that the Clp1-Pcf11 interaction most likely plays a minor role in the functioning of CF IA in these processes. In the light of these results the effects of cellular Clp1 depletion on gene expression have to be interpreted with caution. It seems possible that the absence of Clp1 provoked indirect effects on mRNA synthesis e.g. through interference with the stability of associated proteins, most notably the direct interaction partners Pcf11 and Rna14.

Our analyses did not reveal evidence that would support a functional role for ATP hydrolysis by Clp1. In contrast, several lines of evidence point to a structural role for ATP binding to Clp1. During protein over-expression of mutant Clp1 proteins we consistently found that changes within the P-loop motif gave largely insoluble protein. We interpret these observations such that ATP binding may be required for Clp1 to adopt a properly folded conformation and to escape aggregation. Our CF IA reconstitution experiment suggested furthermore that Clp1 is essential for the assembly of the factor *in vitro*. Assuming that Clp1 indeed acts in CF IA complex formation, why does the K136A T137A mutation, which is deficient in binding to Pcf11, not show any defect in mRNA synthesis or cell growth? One possible answer to this question may be that redundant interactions between subunits exist that cooperate to ensure efficient complex assembly. In support of this idea we observed that Clp1 not only bound to Pcf11 but also to Rna14 (Fig. 2C).

Alternatively, Clp1 may modulate the efficiency of pre-mRNA 3′ end formation in a fashion that is too subtle to be detected in our *in vivo* steady-state gene expression analyses, or in transcription/processing experiments *in vitro*. Since Clp1 is binding to both CF IA and CPF subunits it is tempting to speculate that the protein may relay dynamic interactions and conformational rearrangements that may occur before, during or after the catalysis of cleavage and polyadenylation. It seems particularly intriguing that Clp1 contacts the 3′ endonuclease Ysh1 and that mutant Clp1 proteins bound better than wild-type (Fig. 2C). While this could result from the accidental presentation of an unspecific interaction patch, it may also point to a functionally relevant conformational switch that is associated with the P-loop motif. In such a scenario Clp1 may mediate alternate binding to Pcf11 and Ysh1, respectively. Interestingly, the presence of Pta1 accentuated the preference of Clp1 P-loop mutants to bind to Ysh1. We anticipate that future attempts to further the understanding of Clp1 as a component of the 3′ end formation machinery will have to be performed in the context of an extended interaction network.

## Materials and Methods

### Yeast strains and plasmid constructs

Yeast strains were grown in rich medium (2% bacto-tryptone, 1% yeast extract) supplemented with 2% glucose (YPD) or 2% galactose (YPGal) as indicated in the figures. Strain used in this study were wild-type (MATa ade2 leu2 ura3 trp1-1 his3) and Gal10-UBI-R-HA-CLP1 (MATa ade2 leu2 ura3 trp1-1 his3 TRP1::clp1 [pGAL10-UBI-R-HA-CLP1]). Strains ΔClp1::kan^R^; [pRS423-wt-Clp1], ΔClp1::kan^R^; [pRS423-Clp1 K136A T137A], ΔClp1::kan^R^; [pRS423 yClp1 D161A] were generously provided by Dr. Beate Schwer [15].

### Constructs and site directed mutagenesis

Plasmids used for the expression of recombinant CF IA components for factor reconstitution included previously described GST-Pcf11ΔN288, His6-Rna15/Rna14 and His6-Clp1 (pBD137) constructs [15]. Mutants of the Clp1 protein were generated using the QuickChange mutagenesis kit (Stratagene) and pBD137 as template. Primers were designed and the protocol performed as described in the manufacture's manual. We produced the following mutants: His6-Clp1 K136A-T137A (pSS832) His6-Clp1 D161A (pSS833), His6-Clp1 Q133N (pSH843), His6-Clp1 T137A (pSH844), His6-Clp1 G132P Q133D T135V (loop exchange; pSH845) and His6-Clp1 K136N (pSH828). Constructs expressing Clp1 domains were generated by inserting PCR fragments via oligonucleotide encoded NdeI sites into the same site of pBD137. This generated NTD-Clp1 (amino acids 1–100; pSS837), NTD-CD-Clp1 (amino-acids 1–341; pSS838), CD-Clp1 (amino acids 101–341; pSS839), CD-CTD-Clp1 (amino-acids 101–446; pSS840) and CTD-Clp1 (amino acids 342–446; pSS841). GST fusion proteins were expressed with the use of plasmids GST-Pcf11 (pSH890), GST-Ysh1 (pBD73), GST-Rna14 (pBD221), GST-Rna15 (pUL1043), GST-Yra1 (generously provided by Dr. Françoise Stutz). Constructs for the expression of ProtA-tagged wild-type and mutant Clp1 in yeast were produced by inserting PCR fragments via Nde1-XmaI using oligonucleotide encoded restriction sites into the same sites of vector pNOP1::ProtA-TEV::ADH1 [36]. ProtA-Clp1 (pSH852), ProtA-Clp1K136A-T137A (pUL1044) and ProtA -Clp1 D161A (pSH1047). pGAL10-UBI-R-HA-CLP1 was produced as described [15]. Correct nucleotide sequences were verified by DNA sequencing on both strands.

### Protein expression, CF IA reconstitution and GST pull-down

Expression of recombinant protein and reconstitution of recombinant CF IA was done as previously described [15]. *In vitro* translated proteins were produced using the TNT-coupled transcription-translation system and [^35^S]-methionine according to manufacturers recommendation (Promega). GST pull-downs were performed as previously described [15]. *In vitro* translated proteins were incubated with GST-fusion proteins (100 ng) for 1 h at room temperature. The mixture was bound in a total volume of 860 µl to 20 µl glutathione sepharose (Pharmacia) which was equilibrated in 1 ml PBS, 0.01% NP-40 and 100 µg BSA. The matrix was washed three times with IPP150 (20 mM Tris-HCl pH 8.0, 150 mM KCl, 0.01% NP-40). The proteins were eluted by addition of protein loading buffer and incubation at 95°C. Obtained proteins were separated by SDS-PAGE and visualized by autoradiography.

### RNA extraction and analysis

Total RNA was extracted from yeast strain using the hot phenol method. Northern analyses were carried out as described [15]. PCR products covering the following ORF served as templates for the synthesis of [^32^P]-labeled probes using the random prime labeling kit (Roche): ACT1, ASC1, CYH2, NRD1, TRS31, SER3, SRG1-SER3. Oligonucleotide probes were labeled with [^32^P]-γ-ATP and T4 polynucleotide kinase: anti-18S (CAGACAAATCACTCCA). qRT-PCR analysis was carried out as described [15]. qPCR was done on an Applied Biosystems 7900HT fast real-time PCR system using the SYBR Green PCR Master Mix (Applied Biosystems) according to the manufacturer's instructions.

### Coupled *in vitro* transcription/3′end processing

Reactions were done with extracts depleted of CF IA as previously described [15]. Purification of CF IA factors via ProtA-Clp1 and ProtA-Clp1 K136A T137A was done as described [15]. The final wash step of the purification protocol included variable KCl concentrations (120 mM to 1 M) as indicated in figure 4. For the analysis of 3′ end cleavage activity *in vitro* reactions were done in the absence of radioactive precursors. RNA obtained from *in vitro* transcription/3′ end processing was extracted and subjected to ligation-mediated linker RT-PCR as described [15].

### Nucleotide analysis

To determine the identity of nucleotide bound to CF IA, high-performance liquid chromatography analysis was performed. Prior to sample application, 16 µl of the protein sample was precipitated with 50% of trichloroacetic acid and incubated for 15 min on ice. Subsequently the denatured protein was centrifuged for 10 min at 13'400 rpm on a tabletop centrifuge at 4°C. For neutralization of the supernatant, 10 µl were mixed with 20 µl of a 2 M potassium acetate solution. 20 µl of this solution was applied to a C18 column (ProntoSIL Hypersorb) and developed with 50 mM potassium phosphate buffer (pH 6.8). Nucleotide elution was monitored by absorption at 254 nm and the retention time compared to runs of nucleotide standards. Nucleotide content was calculated by integration of the peak areas.
